# Utilization of Drugs for Attention-Deficit Hyperactivity Disorder Among Young Patients in China, 2010–2019

**DOI:** 10.3389/fpsyt.2021.802489

**Published:** 2022-02-09

**Authors:** Zhiliang Wang, Xiaoyan Wu, Zhenwei Yu, Lingyan Yu

**Affiliations:** ^1^Department of Pediatrics, Shangyu People's Hospital of Shaoxing, Shaoxing, China; ^2^Department of Pharmacy, Sir Run Run Shaw Hospital, Zhejiang University School of Medicine, Hangzhou, China; ^3^Department of Pharmacy, Second Affiliated Hospital, Zhejiang University School of Medicine, Hangzhou, China

**Keywords:** methylphenidate, atomoxetine, pediatric, adolescent, prescription

## Abstract

**Objective:**

The use of attention-deficit hyperactivity disorder (ADHD) medication is increasing worldwide, but its status in China is unknown. This research aimed to assess the trends of ADHD medication use in young Chinese patients between 2010 and 2019.

**Methods:**

Dispensing data related to ADHD medication use were extracted from the Hospital Prescription Analysis Cooperative Project of China. The trends in the yearly prescription number of ADHD drugs and corresponding cost were analyzed. We further stratified the data by age, sex, and specific drug.

**Results:**

From 2010 to 2019, sampled prescriptions for ADHD medication increased from 902 to 4531, and the total expenditure increased rapidly from 276,580 to 2,412,308 Chinese Yuan. Prescriptions for males were almost fourfold more than that for females. Patients aged 6–11 years had the highest number of prescriptions for ADHD medication each year, accounting for more than 50% of the total number of prescriptions. The percentage of methylphenidate prescriptions decreased from 91.9% in 2010 to 76.9% in 2019, and the corresponding cost declined from 77.3% to 66.8%. In contrast, atomoxetine prescriptions increased progressively and accounted for about 24.5% of the total prescriptions at the end of the study.

**Conclusions:**

The use of ADHD drugs and the corresponding cost increased rapidly in China, and methylphenidate was the most frequently prescribed medicine. The increase in ADHD prescriptions requires attention to ensure that it reflects appropriate use, especially in patients aged 6–11 years.

## Introduction

Attention deficit hyperactivity disorder (ADHD) is one of the most common mental disorders in childhood and adolescence (1). It is a group of syndromes characterized by persistent symptoms of inattention, hyperactivity, and impulsivity, which are not commensurate with the individual's level of development (2). Many symptoms persist into adulthood, negatively affecting patients, their families, and society. It is more prevalent in males than in females, particularly in school-age children (3). The etiology of ADHD is presently unclear. Pharmacotherapy is an effective therapeutic option for the management of ADHD symptoms and impairments (4, 5). In the last few decades, an increased use of ADHD medications has been observed in several countries, mainly in Europe and North America (3, 4). However, data are limited regarding the prevalence and treatment patterns in non-Western countries (6). Therefore, this study was conducted to evaluate the prescribing status and trends of ADHD drugs in China for a decade.

## Methods

This study was approved by the Ethics Committee of the Second Affiliated Hospital, Zhejiang University School of Medicine. The requirement for informed consent was waived owing to the retrospective nature of the study.

### Data Source

Data were collated using the Hospital Prescription Analysis Cooperative Project database, which has been previously validated and extensively used for epidemiological studies in China (7–12). The database contains participating hospitals' prescription information for 40 random days per year. The prescription data include the unique prescription code, sex, age, date, location, hospital code, diagnosis, drug generic name, and price of drugs; however, the owner of the prescription was not identified.

### Data Extraction and Analysis

Hospitals located in six major regions of China (Beijing, Shanghai, Hangzhou, Guangzhou, Tianjin, and Chengdu) that participated in the program continuously from 2010 to 2019 were identified. All prescriptions for ADHD drugs for outpatients diagnosed with ADHD were identified and extracted for analysis. The currently available ADHD drugs in China are methylphenidate (N06BA04) and atomoxetine (N06BA09); this study only focuses on these two drugs. The study focused on prescriptions that were written for participants in the 2–24 year age group. The number of yearly prescriptions was calculated by adding up the prescriptions on the sampled days in each year. The yearly expenditure was calculated by adding up all the costs of the ADHD medications. It should be noted that yearly prescriptions and costs in this study corresponded to 40 sampled days in each year. The trends of yearly prescriptions and cost were assessed and further stratified by age and sex of patients on the prescriptions and the specific drug. Trends in proportions were tested by log-linear analysis. Other trends were analyzed by means of the Mann–Kendall trend test. The criterion for statistical significance was a two-sided *P* value of.05. All the statistical analyses were run in R (V 3.5.0, http://www.R-project.org).

## Results

### Overall Trends in ADHD Medication Use and Expenditure

**Figure 1:**
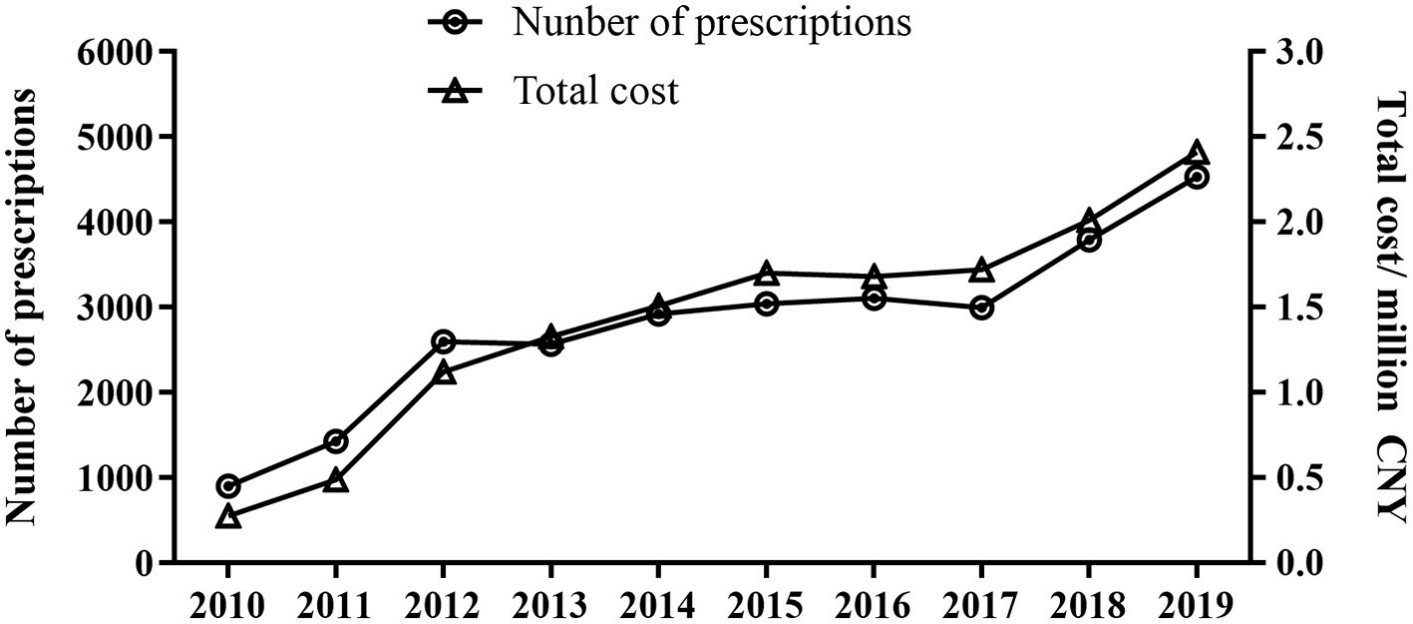
Total trends of prescriptions and expenditure of ADHD drugs from 2010 to 2019.

A total of 27,882 prescriptions written from 2010 to 2019 from 14 hospitals were included in the study. As shown in Figure 1, the overall sampled ADHD prescriptions increased from 902 in 2010 to 4531 in 2019 (*P* < 0.001), and the total expenditure increased rapidly from 276,580 in 2010 to 2,412,308 Chinese Yuan (CNY) in 2019 (*P* < 0.001).

**Figure 2:**
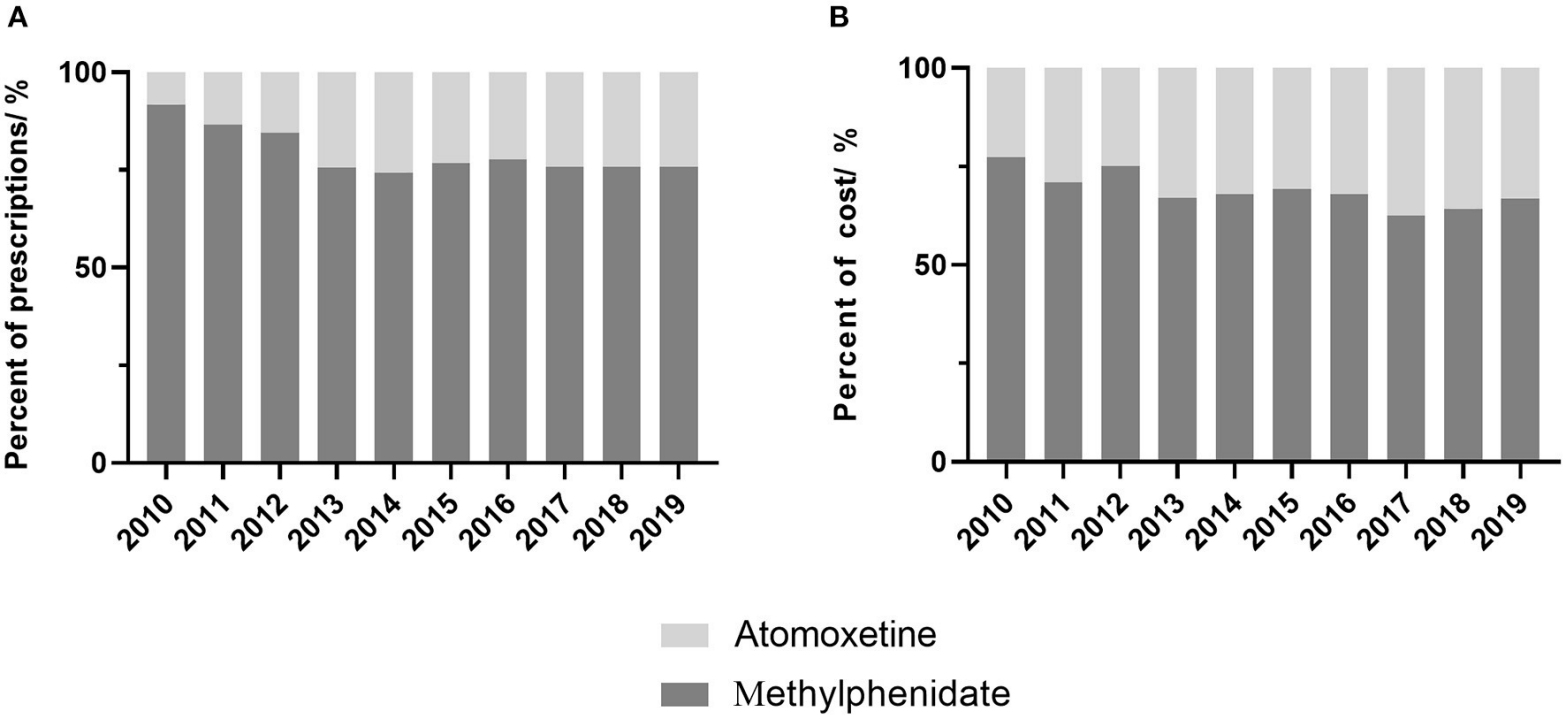
Proportion of prescriptions and cost of ADHD drugs by year. **(A)** proportion of prescriptions, **(B)** proportion of cost.

The percentage of prescriptions containing methylphenidate decreased from 91.9% in 2010 to 76.9% in 2019 (*P* = 0.013), and the corresponding cost declined from 77.3% to 66.8% (*P* = 0.005). Atomoxetine accounted for 33.2% of the cost and about 24.5% of the total prescriptions by 2019 (Figure 2).

### ADHD Drug Use by Age and Sex

Among all prescriptions for ADHD drugs, the major percentage comprised patients aged 6–11 years, and this percentage continued to increase during the study period (Table 1). The number of prescriptions for patients aged 12–18 years increased; however, eventually the percentage decreased (*P* < 0.001 and *P* = 0.002, respectively). Prescriptions for males were about 80% of all prescriptions in each year, and no significant trend was noted (Table 1, *P* = 0.957).

**Table 1:** Characteristics of patients with ADHD prescriptions between 2010 and 2019.

	**2010**	**2011**	**2012**	**2013**	**2014**	**2015**	**2016**	**2017**	**2018**	**2019**
**Age (years)**										
2–5	8 (0.89)	8 (0.56)	7 (0.27)	16 (0.62)	4 (0.14)	5 (0.16)	9 (0.29)	9 (0.30)	9 (0.24)	13 (0.29)
6–11	550 (60.98)	921 (64.50)	1,770 (68.21)	1,739 (67.80)	2,030 (69.43)	2,116 (69.58)	2,159 (69.51)	2,035 (67.90)	2,688 (70.87)	3,255 (71.84)
12–18	334 (37.03)	473 (33.12)	788 (30.37)	779 (30.37)	858 (29.34)	902 (29.66)	913 (29.39)	915 (30.53)	1,061 (27.97)	1,221 (26.95)
19–24	10 (1.11)	26 (1.82)	30 (1.16)	31 (1.21)	32 (1.09)	18 (0.59)	25 (0.80)	38 (1.27)	35 (0.92)	42(0.93)
**Sex**										
Male	723 (80.2)	1,174 (82.2)	2,158 (83.2)	2,105 (82.1)	2,358 (80.6)	2,490 (81.9)	2,543 (81.9)	2,437 (81.3)	723 (80.2)	1,174 (82.2)
Female	179 (19.8)	254 (17.8)	437 (16.8)	460 (17.9)	566 (19.4)	551 (18.1)	563 (18.1)	560 (18.7)	179 (19.8)	254 (17.8)

*Data are presented as prescriptions (percentage%)*.

**Figure 3:**
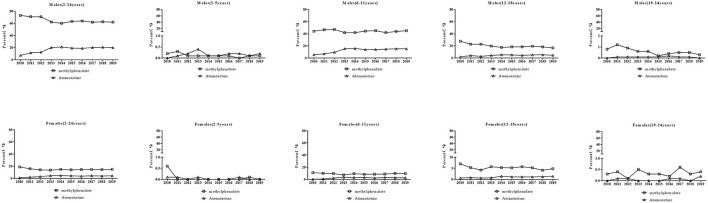
ADHD drug use stratified by age and sex.

The specific ADHD drugs used in each age group and sex are shown in Figure 3. For methylphenidate, the prescriptions for male patients decreased from 73.1% to 62.0% (*P* = 0.011), especially in boys aged 12–18 years (*P* = 0.001). There was no change in the percentages of prescriptions over time for boys aged 2–5 years (*P* = 0.19) and 6–11 years (*P* = 0.483). Prescriptions for boys aged 19–24 years were associated with a minor portion and exhibited a decreasing trend (*P* = 0.011). Conversely, there was no significant trend in the percentages of prescriptions over time for female patients although the percentage in 2019 (14.9%) was lower than that in 2013 (18.8%) (*P* = 0.163). There was no significant trend in the prescriptions for females aged 2–5, 6–11, and 19–24 years (all *P*s>0.05). The percentage of prescriptions for females aged 12–18 years decreased (*P* = 0.003). For atomoxetine, an increase in the percentages of prescriptions for male patients (7.2% to 20.2%) during the study period was observed (*P* = 0.007). The increasing trend was also observed in prescriptions for boys aged 6–11 (*P* < 0.001) and 12–18 years (*P* = 0.021). There was no change in the percentage of prescription for males aged 2–5 years (*P*>0.05) and 19–24 years (*P*>0.05) over time. An increase in the percentage of prescriptions for females (1.0% to 4.4%) was observed (*P* = 0.017) similar to that in prescriptions for girls aged 6–11 years (*P* = 0.034) and 12–18 years (*P* = 0.005). There was no change in the percentages of prescriptions for girls aged 2–5 years (*P*>0.05) and 19–24 years (*P*>0.05).

## Discussion

To the best of our knowledge, this is the first study to assess the trends of ADHD drug use in young outpatients in China. This study found that both prescriptions and the corresponding cost for ADHD drugs increased significantly from 2010 to 2019 and that the predominant drug for ADHD was methylphenidate.

Similar to other countries, a dramatic increase in prescriptions and cost were found in this study (13, 14). The possible reasons for the rise in the number of prescriptions are multifactorial, including an increase in recognition and diagnosis. Parental desires to improve their child's academic achievements may lead to an increase in prescription rates (15). A recent study revealed that ADHD symptoms showed significant improvements for patients on placebos, and the placebo effect may have a negative effect on prescription rates for patients with ADHD (16). As the data were collected from hospitals in six regions on 40 random days of each year, it is difficult to extrapolate this information to a whole year or an entire population. However, given a stable prevalence of ADHD in recent years and the continuous increase in prescriptions, unmet needs are deemed present. Thus, efforts should be made to ensure that all patients with ADHD who need medication are treated. It is noteworthy that the yearly cost of ADHD drugs in 2019 was more than eightfold higher than that in 2010; however, the yearly prescriptions only increased fivefold.

Prescriptions for patients aged 6–18 years comprised the majority of ADHD medication use in China. This is similar in other countries (17, 18). However, the number and percentages of prescriptions for this age group both increased progressively, which prompts special attention directed at improving the management of ADHD. National Institute for Health and Care Excellence guidelines do not recommend drug treatment with ADHD medications in patients aged under 6 years (19). Prescriptions for this age group are few, and clinical practice appears to follow these guidelines well. This study showed that prescriptions for male patients with ADHD are fourfold greater than those for females, and this result is in accordance with another study on prevalence (3, 17, 18, 20).

In terms of drug therapy, stimulants, such as methylphenidate, are considered the first-line pharmacological treatment for ADHD and have been used for the last 50 years. In this study, methylphenidate was also the most frequently prescribed ADHD medication. Whereas the mechanism by which it reduces symptoms in ADHD is not completely clear, it is believed that it enhances intrasynaptic concentrations of dopamine and noradrenaline in the frontal cortex as well as in the subcortical brain regions associated with motivation and reward. The common adverse effects of methylphenidate are appetite loss, abdominal pain, headaches, and sleep disturbance (21). Furthermore, clinicians are encouraged to monitor heart rate and blood pressure during its use (22).

Another commonly used drug for ADHD in China is atomoxetine, which is used as a second-line treatment (19). Presently, most people believe that the therapeutic effect of this drug is related to the selective inhibition of norepinephrine reuptake by means of presynaptic amine pumps (23). This inhibition can enhance the reversal effect of norepinephrine, thus improving the symptoms of ADHD and indirectly promoting cognitive completion, attention, and concentration (24). Adverse effects of atomoxetine include initial somnolence and gastrointestinal tract symptoms, particularly if the dosage is increased too rapidly.

One study shows that both methylphenidate and atomoxetine were effective in improving a wide range of emotional/behavioral problems in youths with ADHD. However, the methylphenidate group correlated with a greater improvement in aggressive behavior, somatic complaints, and conduct problems (25).

Our study has certain limitations. The database only included the prescriptions of patients with ADHD, but the clinical profiles, including the severity or treatment outcome, were not measured and should be investigated in further studies. Sampling bias may exist because this study only involved hospitals located in six regions of China.

## Conclusion

This study provides the most recent national data on pediatric use of ADHD medications in China. The number of prescriptions for ADHD medications and the costs were found to increase rapidly. We, therefore, need to monitor trends of drug use with regard to ADHD medications and determine their safety in children with ADHD.

## Data Availability Statement

The original contributions presented in the study are included in the article/supplementary material, further inquiries can be directed to the corresponding author/s.

## Ethics Statement

The studies involving human participants were reviewed and approved by Ethics Committee of Second Affiliated Hospital, Zhejiang University School of Medicine. Written informed consent from the participants' legal guardian/next of kin was not required to participate in this study in accordance with the national legislation and the institutional requirements.

## Author Contributions

LY: conceptualization, visualization, resources, and writing of the review and editing. ZY and LY: methodology. ZW and LY: data curation. ZW, XW, ZY, and LY: formal analysis. ZW and XW: investigation. LY and XW: validation. ZW, XW, and ZY: writing of the original draft.

## Conflict of Interest

The authors declare that the research was conducted in the absence of any commercial or financial relationships that could be construed as a potential conflict of interest.

## Publisher's Note

All claims expressed in this article are solely those of the authors and do not necessarily represent those of their affiliated organizations, or those of the publisher, the editors and the reviewers. Any product that may be evaluated in this article, or claim that may be made by its manufacturer, is not guaranteed or endorsed by the publisher.
